# Regulation of Selenocysteine Content of Human Selenoprotein P by Dietary Selenium and Insertion of Cysteine in Place of Selenocysteine

**DOI:** 10.1371/journal.pone.0140353

**Published:** 2015-10-09

**Authors:** Anton A. Turanov, Robert A. Everley, Sandra Hybsier, Kostja Renko, Lutz Schomburg, Steven P. Gygi, Dolph L. Hatfield, Vadim N. Gladyshev

**Affiliations:** 1 Division of Genetics, Department of Medicine, Brigham and Women’s Hospital and Harvard Medical School, Boston, Massachusetts, 02115, United States of America; 2 Department of Cell Biology, Harvard Medical School, Boston, Massachusetts, 02115, United States of America; 3 Institute for Experimental Endocrinology, Department of Urology, Charité-Universitätsmedizin Berlin, Berlin, Germany; 4 Molecular Biology of Selenium Section, Mouse Cancer Genetics Program, Center for Cancer Research, National Cancer Institute, National Institutes of Health, Bethesda, Maryland, 20892, United States of America; Robert Gordon University, UNITED KINGDOM

## Abstract

Selenoproteins are a unique group of proteins that contain selenium in the form of selenocysteine (Sec) co-translationally inserted in response to a UGA codon with the help of *cis-* and *trans*-acting factors. Mammalian selenoproteins contain single Sec residues, with the exception of selenoprotein P (SelP) that has 7–15 Sec residues depending on species. Assessing an individual’s selenium status is important under various pathological conditions, which requires a reliable selenium biomarker. Due to a key role in organismal selenium homeostasis, high Sec content, regulation by dietary selenium, and availability of robust assays in human plasma, SelP has emerged as a major biomarker of selenium status. Here, we found that Cys is present in various Sec positions in human SelP. Treatment of cells expressing SelP with thiophosphate, an analog of the selenium donor for Sec synthesis, led to a nearly complete replacement of Sec with Cys, whereas supplementation of cells with selenium supported Sec insertion. SelP isolated directly from human plasma had up to 8% Cys inserted in place of Sec, depending on the Sec position. These findings suggest that a change in selenium status may be reflected in both SelP concentration and its Sec content, and that availability of the SelP-derived selenium for selenoprotein synthesis may be overestimated under conditions of low selenium status due to replacement of Sec with Cys.

## Introduction

Selenium (Se) is an essential microelement [1,2,3]. In mammals, it is present primarily within selenoproteins in the form of selenocysteine (Sec) [4]. Sec is encoded by a UGA codon and becomes inserted into selenoproteins by a set of specific *cis*- and *trans*-acting factors. The human selenoproteome is encoded by 25 selenoprotein genes [5], and the majority of selenoproteins with known functions are oxidoreductases, in which Sec is located in catalytic sites [6].

One mammalian selenoprotein, selenoprotein P (SelP, SEPP1), is different from other selenoproteins in that it has multiple Sec residues [7]. This protein is composed of an N-terminal thioredoxin domain containing one Sec and a C-terminal Sec-rich region containing from 6 (e.g., in the naked mole rat) to 14 (e.g., in the dog) Sec residues [8]. SelP is primarily synthesized by hepatocytes, secreted from liver and is the most abundant selenoprotein in plasma [9,10]. SelP delivers Se, in the form of Sec, to other organs with receptors, such as ApoER2 and megalin, that support SelP uptake [7]. The overall effect of having SelP is Se retention and redistribution in the body. SelP-deficient mice are characterized by Se deficiency and can be rescued by excess dietary Se [7,9].

SelP emerged as a biomarker of human Se status, because it is regulated by dietary Se within a broad range of Se levels, and also because this selenoprotein can be readily assayed in human plasma [11,12,13]. SelP also has a high Sec content and serves a key role in organismal selenium homeostasis. The expression of SelP and its levels in human plasma were assumed to be directly proportional to Se status following ingestion of normal and low amounts of dietary Se. However, quantification of Se in SelP containing 10 Sec codons yielded a selenium content of only 7.5 +/- 1.0 atoms/polypeptide in rat [14]. It has been unclear whether this decreased selenium content is due to imperfect translation, premature translational termination, a loss of selenium following protein synthesis or for other reasons.

In the current study, we found that Cys may be inserted in place of various Sec residues in SelP. Under normal dietary intake, up to 8% of Sec residues are replaced with Cys in human SelP, whereas in cell culture, the Sec/Cys ratio at the Sec sites is regulated by both Se status and concentration of thiophosphate, leading to the Sec/Cys ratio from nearly zero to nearly one. We discuss these data with regard to a possible overestimation of SelP-derived Sec availability for selenoprotein biosynthesis under low Se status.

## Materials and Methods

### Reagents

HepG2 human hepatoma cells were from ATCC. Sodium selenite (Na_2_SeO_3_), sodium thiophosphate (SPO_3_; formula Na_3_SPO_3_), iodoacetamide and DTT were from Sigma. Pierce HisPur chromatography cartridge and monoclonal anti-hSelP antibodies were from Thermo Scientific. Unless otherwise stated, all remaining reagents were from Sigma. ^75^Se isotope ([^75^Se]selenious acid (specific activity, 1,000 Ci/mmol) was purchased from the Research Reactor Facility, University of Missouri (Columbia, MO).

### Metabolic labeling of cells with ^75^Se, and supplementation of cells with Se and thiophosphate

HepG2 cells were grown in high glucose DMEM supplemented with 10% fetal bovine serum and antibiotic/antimycotic solution (all from Life Technology) at 37°C in 5% CO_2_ in 150 cm^2^ flasks. Cells were split 1:4 the day before metabolic labeling in 10 cm dishes. Cells were incubated for 24 h, then washed two times with PBS, and culture medium replaced with DMEM without fetal bovine serum in the presence or absence of 100 nM of sodium selenite or 1 mM of thiophosphate (final concentration), and labeled or not for 24 h with 10 **μ**Ci of ^75^Se freshly neutralized with NaOH. Cells with only DMEM served as a control. Conditioned media and cells were collected separately. Media samples were concentrated in Amicon Ultra centrifugal filter units with 10 kDa cut-off (Millipore). Cells were then resuspended in PBS and sonicated. 30 μg of total soluble protein from each cell lysate sample were resolved by SDS-PAGE and transferred onto a PVDF membrane (Life Technology). Selenoprotein patterns were visualized with a PhosphorImager. For large-scale SelP preparation, HepG2 cells were incubated in 15 cm dishes in DMEM without serum with addition of 100 nM sodium selenite or 1 mM of thiophosphate or with medium only. After 24–48 h, media samples were replaced with fresh medium or the medium containing sodium selenite or thiophosphate. Conditioned media from different incubations were pulled together resulting in about 250 ml of medium for each sample.

### Isolation of human SelP from cell culture media

**Fig 1:**
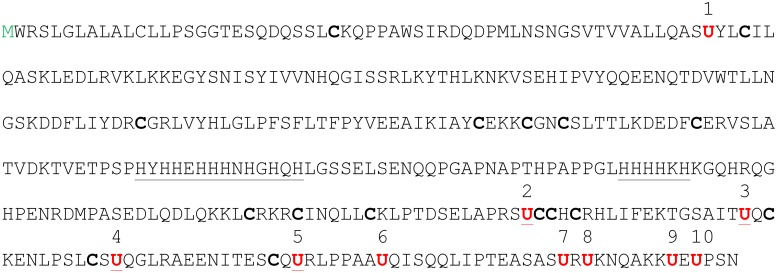
Human SelP sequence. Positions of the ten Sec residues are indicated by numbers and shown in red. Positions of Cys residues are highlighted in bold. Natural His-rich segments, which allow affinity isolation of human SelP, are underlined.

Secreted SelP was purified from HepG2 cell culture media using metal affinity chromatography utilizing His-rich segments of SelP (Fig 1) as described [15]. Briefly, media samples were filtered thought 0.2 **μ**m filter and concentrated using Amicon Ultra centrifugal filters to a final volume of ~10 ml. Next, media samples were equilibrated with loading buffer and SelP was purified on HisPur 2 ml columns according to the manufacturer’s manual. Purification process was monitored by SDS-PAGE and Western blotting with anti-SelP antibodies. Imidazol elution fractions were collected, dialyzed against PBS for 12 h, then concentrated to a final volume of 100–200 **μ**l.

### Isolation of human SelP from plasma

SelP was isolated from human plasma by affinity purification. A SelP-specific monoclonal antibody (2.6 mg, anti-SePP#7h6, ICI—immunochemical intelligence GmbH, Berlin, Germany) was coupled to AminoLink® Plus Coupling Gel (2 mL bed volume, Pierce Biotechnologies, Inc., Rockford, IL). The protocol was approved by the ethics committee of the Charité-University Medicine Berlin (EA2/103/10) for healthy human probands, and we obtained the written consent of the five donors used. The five human plasma samples (50 ml each) were pooled and mixed with one volume of PBS, pH 8.0. Complete protease inhibitor cocktail tablets (n = 10, Roche Applied Science) were added, the mixture was centrifuged at 1248×g (3000 rpm) for 10 min at 4°C, and consecutively filtered through 5 **μ**m, 0.8 μm and finally 0.2 μm filters (Schleicher & Schuell, Dassel, Germany). The filtrate was loaded by gravity flow onto the affinity column and washed with PBS, pH 8.0, until the OD_280_ of the flow-through was < 0.005. SelP was eluted with citric acid, pH 2.0, in 15 fractions of 1 ml each. Each fraction was supplemented by 50 **μ**L 1 M Tris, pH 8.8. The fractions with highest OD_280_ readings were combined (eluates E2-E6), aliquoted and stored at -80°C. Aliquots of the initial, flow-through and eluate samples were analyzed in a luminescence-based in-house immunoassay with two monoclonal mouse anti-human SelP antibodies. The detection antibody was labeled with an acridinium ester. 26% of the SelP sample-load was recovered in the eluates.

### Liquid chromatography (LC)-MS/MS

Purified SelP samples were reduced with DTT, followed by alkylation of Cys and Sec residues with iodoacetamide as described [16]. Alkylated proteins were resolved by SDS-PAGE using Novex NU-PAGE system (Life Technology) and stained with Coomassie blue. Protein bands corresponding to SelP (individual samples representing the conditions analyzed in the current study) were cut out and subjected to in-gel tryptic digestion and LC-MS/MS analysis. In-gel trypsin digestion of the destained protein bands was carried out for 14 h at 37°C. The resulting peptide mixture was extracted from the gel slices and loaded into a fused silica microcapillary packed with Magic C18AQ beads (Michrom Bioresources). Reversed phase liquid chromatography was performed using an Agilent 1100 pump and a Famous autosampler (LC Packings). Peptides were eluted from the column with a 90-min acetonitrile gradient and detected using an LTQ-Orbitrap Velos (Thermo-Fisher Scientific).

### Database analysis and quantification

MS/MS spectra were analyzed against a concatenated IPI_Human database (version 3.60) (http://www.ebi.ac.uk/IPI/) using the Sequest algorithm (Version 28, Thermo-Fisher Scientific) and a 0.5% false discovery rate. Database search criteria were as follows: two missed cleavages, a precursor mass tolerance of 50 ppm, an MS/MS fragment ion tolerance of 0.8 Da, and the following variable modifications: oxidation (M), deamidation (NQ), and alkylation on Cys and Sec. Peptide abundances were calculated using the area under the curve representing the entire chromatographic peak of interest. The area under the curve for each Cys and Sec containing form was divided by the sum of both forms to calculate a percent of the total peptide signal, as previously carried out for thioredoxin reductase 1 [16].

## Results

### Characterization of SelP secreted by HepG2 cells

**Fig 2:**
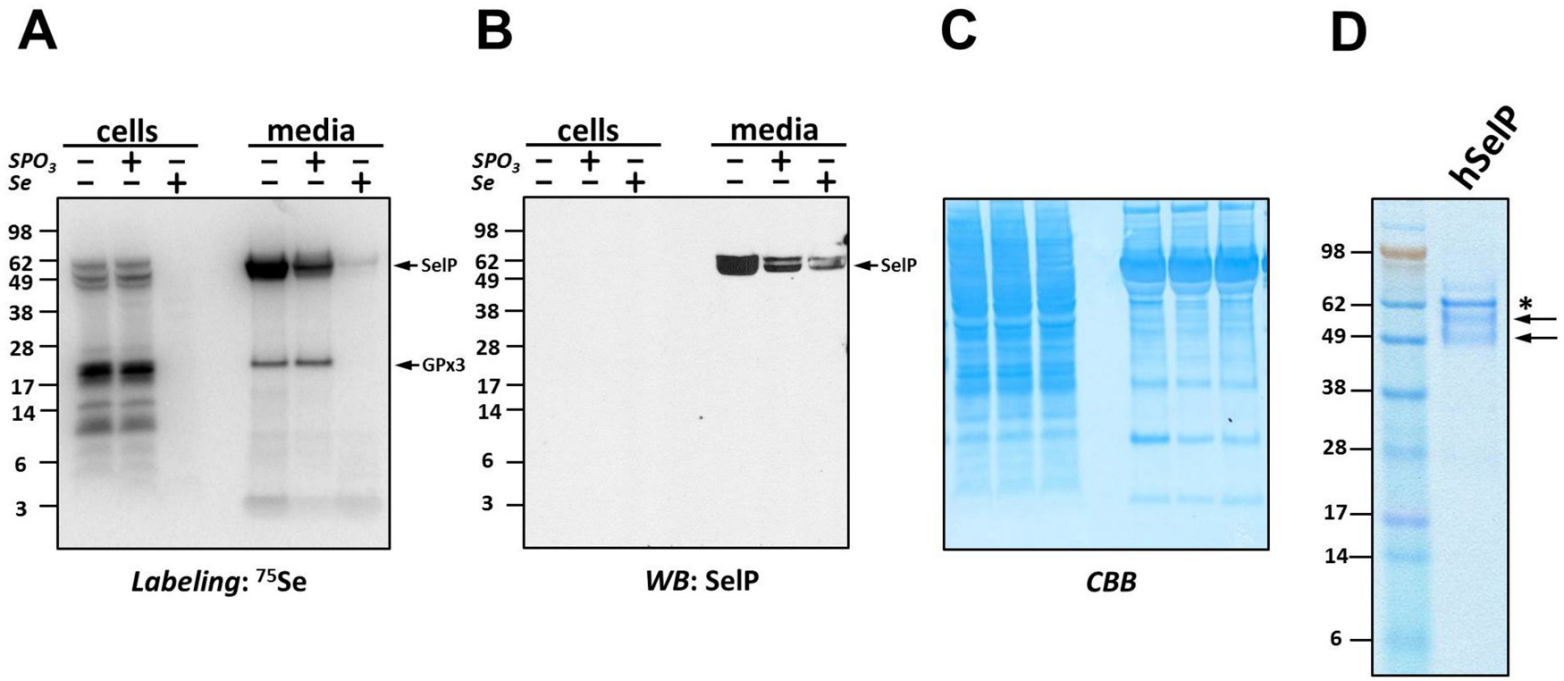
SelP expression in hepatocytes and its regulation by selenium and thiophosphate. (A) HepG2 cells were cultured without or with 100 nM Se or 1 mM thiophosphate (SPO3), metabolically labeled with ^75^Se, and cell lysates and culture media samples analyzed by SDS-PAGE and PhosphorImager for selenoprotein expression. Positions of SelP and GPx3 are indicated by *arrows* on the right, and protein molecular weight markers are indicated on the left. (B) Samples from panel *A* were analyzed by Western blotting (WB) with anti-SelP antibodies. Positions of protein molecular weight markers are indicated on the left and SelP is indicated by *arrows* on the right. (C) Coomassie blue (*CBB*) staining of samples from *A* and *B*, used as a loading control. (D) SelP isolated from human blood was analyzed by SDS-PAGE and stained with Coomassie blue. Protein molecular weights markers in kDa are shown on the *left*. Positions of different SelP forms are indicated by *arrows*. Position of serum albumin is indicated by an *asterisk*.

Human SelP has two His-rich segments (Fig 1), which can be employed for isolation of native SelP using metal affinity chromatography [15]. As SelP is largely expressed in hepatocytes and secreted into the blood stream [9,10,16,17], we used human hepatoma HepG2 cells as a source of SelP. Cells were grown in DMEM, or in medium supplemented with 100 nM sodium selenite (as a source of Se) or 1 mM thiophosphate (SPO_3_), and expression of SelP and other selenoproteins was analyzed by metabolic labeling of cells with ^75^Se (Fig 2A). This experiment revealed a ^75^Se labeling pattern typical of mammalian cells. As expected, these cells secreted ^75^Se-labeled SelP, and we also found that they secreted glutathione peroxidase 3 (GPx3). SelP was the most abundant secreted selenoprotein. The presence of SelP in media samples was confirmed by Western blotting with anti-SelP antibodies (Fig 2B). As expected, SelP was not detected in cells, because it was rapidly secreted following maturation and accumulated in cell culture media. The method allowed distinguishing SelP from cellular selenoproteins, such as thioredoxin reductase 1, which exhibit similar migration properties on SDS-PAGE gels.

We have shown previously that 1 mM thiophosphate did not significantly affect the growth of NIH 3T3 cells, but could dramatically influence Sec insertion into thioredoxin reductases [16]. Interestingly, 100 nM sodium selenite dramatically decreased incorporation of the radioactive tracer into selenoproteins, apparently due to competition of “cold” Se with ^75^Se (Fig 2A). Despite inefficient labeling of cells in the presence of sodium selenite, SelP was detected by Western blotting (Fig 2B). This analysis also revealed that SelP secreted from HepG2 cells was present in two major forms differing in molecular weight, apparently due to differential N-glycosylation levels, as shown previously [18,19]. Gels were stained with Coomassie blue as loading control (Fig 2C). We carried out isolation of SelP secreted from HepG2 cells using metal affinity chromatography, which revealed significant enrichment of this protein, represented by two bands (see below). A similar two-band pattern was observed for SelP isolated from human plasma (Fig 2D). Subsequent MS/MS analysis of proteins isolated from gels revealed that both bands contained full-length human SelP. Overall, these analyses demonstrated robust expression of SelP by HepG2 cells and its amenability to affinity chromatography.

**Fig 3:**
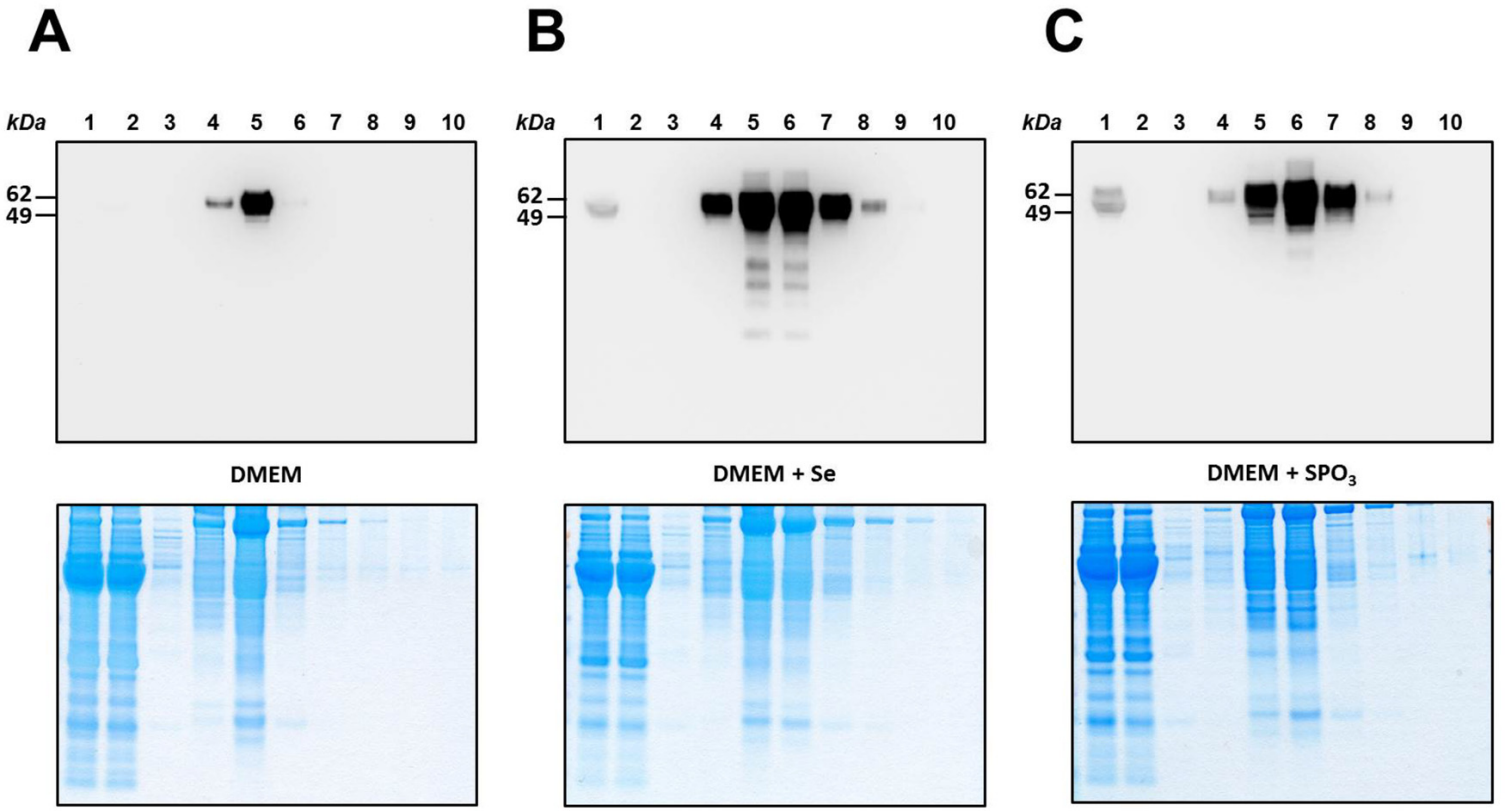
Isolation of SelP from culture media of HepG2 cells. Medium samples from HepG2 cells were chromatographed on HisPur resin. Fractions were analyzed by SDS-PAGE, the gels stained with Coomassie blue (*Lower panels*) and subjected to Western blotting with anti-SelP antibodies (*Upper panels*). *Lane 1*, initial sample, *lane 2*, flow-though fraction, *lane 3*, buffer wash, *lane 4–10*, elution with a gradient of imidazole from 0 to 200 mM in loading buffer. (A) HepG2 cells grown on DMEM medium only (control). (B) HepG2 cells grown on DMEM supplemented with 100 nM Se. (C) HepG2 cells grown on DMEM supplemented with 1 mM thiophosphate (SPO_3_). Protein molecular weights markers in kDa are shown on the *left*. Experimental details are given in *Materials and Methods*.

For large-scale preparation of SelP from HepG2 cells, approximately 250 ml of conditioned DMEM media were collected for each experimental condition. Filtered and concentrated media samples were subjected to metal-affinity purification using natural His-rich segments of SelP. Purification was monitored by Western blotting with anti-SelP antibodies (Fig 3, upper panels). Fractions containing SelP were pulled together and analyzed by LC-MS/MS. Protein flow through was assessed by Coomassie blue staining (Fig 3, lower panels). The conditions of small- and large-scale isolations were slightly different, so the patterns of elution cannot be directly compared, but both procedures worked sufficiently well to enrich SelP for subsequent analyses.

### Selenite and thiophosphate modulate Sec to Cys ratio at multiple positions in SelP

We previously found that Cys can be inserted in place of Sec in thioredoxin reductases (TRs) in a process that required a UGA codon and a SECIS element [15]. In addition, thiophosphate could replace selenophosphate as a substrate for Sec synthase (SecS), an enzyme responsible for the final step of Sec biosynthesis [20]. To examine the possibility that Cys replaces Sec in SelP, this protein was isolated from control, selenite-supplemented and thiophosphate-supplemented cells and subjected to LC-MS/MS analysis, examining all ten Sec positions in human SelP. Sample quality and content allowed quantitative estimation of Sec to Cys replacement at Sec positions three, four and five (Table 1); other positions showed low spectral count precluding quantitative assessment. Depending on position, 0–18% Cys were found in Sec positions. The difference among peptides is likely due to the difference in detection of various peptides by mass spectrometry, so these numbers should be viewed as estimated levels of Sec/Cys insertion. SelP isolated from cells grown in the presence of 100 nM selenite showed only Sec in Sec positions. In contrast, SelP from cells maintained in the presence of thiophosphate showed nearly complete replacement of Sec with Cys in the identified peptides (Table 1). Thus, the Sec/Cys ratio was regulated by both Se availability and the levels of thiophosphate in cell culture medium.

**Table 1:** Tryptic Sec-containing peptides of SelP isolated from HepG2 media samples.

Sample	Tryptic Peptide	Cys, %	Sec, %
Control	TGSAIT**U**QCK	0	100
	ENLPSLCS**U**QGLR	0	100
	AEENITESCQ**U**R	18.2	81.8
Selenite	TGSAIT**U**QCK	0	100
	ENLPSLCS**U**QGLR	0	100
	AEENITESCQ**U**R	0	100
Thiophosphate	TGSAIT**U**QCK	100	0
	ENLPSLCS**U**QGLR	100	0
	AEENITESCQ**U**R	95.5	4.5

Percent Sec and Cys in SelP peptides was assessed by MS/MS as described in Materials and Methods.

### Sec and Cys insertion in native human SelP

Our previous data revealed that TR1 isolated from mouse liver had 10% Cys in place of Sec, even under conditions when mouse diets were supplemented with 0.1 ppm Se [16]. To examine Cys insertion into native SelP, we analyzed Sec to Cys replacement in SelP isolated from human donor plasma (S1 Fig). Here, Sec and Cys could be quantified at Sec positions two, three, four and five (Table 2). Interestingly, Cys insertion was unequal among Sec positions. Depending on position, up to 8% Sec residues were replaced with Cys, with an average of 4.1% Cys in place of Sec. Thus, Cys is present naturally in SelP under common dietary conditions.

**Table 2:** Tryptic Sec-containing peptides of SelP isolated from pooled (n = 5) human plasma.

Sec position	Tryptic Peptide	Sec, %	Cys, %
2	S**U**CCHCR	100	0
3	TGSAIT**U**QCK	91.5	8.5
4	ENLPSLCS**U**QGLR	97.9	2.1
5	AEENITESCQ**U**R	98.3	1.7

Percent Sec and Cys in SelP peptides was assessed by MS/MS as described in Materials and Methods.

## Discussion

Sec insertion at UGA codons is known to be at competition with termination of protein synthesis [1]. However, the levels of full-length selenoproteins are often viewed as being equivalent with their selenium content. Although quantification of Se content in selenoproteins pointed out that it is lower than one atom per Sec site per selenoprotein, it has been assumed that a fraction of Se is lost from selenoproteins under physiological conditions or during protein preparation. However, we recently showed that Cys can be inserted into TR1 and that up to 10% Sec sites are occupied by Cys in this selenoprotein in mice fed common rodent chow containing normal amounts of dietary selenium [16].

A key finding of the present study is that SelP also contains Cys in place of Sec. This is particularly relevant since SelP is a protein that largely regulates organismal Se homeostasis. This selenoprotein of exceptional significance has emerged as a biomarker of Se status based on both animal and human studies. SelP has also been suggested to be a biomarker for prostate cancer [21,22]. Previous studies revealed the Se content of only 7.5 +/- 1.0 atoms/SelP polypeptide that contains 10 predicted Sec sites [14], consistent with the idea that at least some of the previous discrepancy between the predicted and observed Se content of the protein may be explained by Cys insertion at the Sec sites.

Depending on the location of Sec within SelP, up to 8% of Sec sites were occupied by Cys in the selenoprotein isolated from plasma of five healthy donors (S1 Fig), with the average of 4% Cys across Sec positions. Spectral counts were sufficient to quantify only 4 sites in SelP. Thus, these numbers represent approximate Cys content of Sec at specific sites. We further found that this Sec/Cys ratio can both decrease and increase depending on the availability of Se and the selenophosphate antagonist, thiophosphate, because treatment of HepG2 hepatocytes with thiophosphate completely reversed the ratio such that only Cys was detected at certain Sec positions. This is also consistent with the previously observed regulation of Cys insertion at the Sec site in TR1. The cell culture conditions used in the current study represent the proof of principle with regard to Cys insertion at Sec positions in SelP, but do not correspond to physiological conditions, especially when high levels of thiophosphate were used. However, these studies provide a foundation for further analyses of Cys and Sec in SelP isolated from human subjects.

The finding of Cys at Sec positions in a key regulator of organismal Se homeostasis is noteworthy, as Se status may be influenced by dietary status, disease, age and other factors. Thus, Se availability may be affected, not only at the levels of SelP, but also at the level of Sec insertion into this protein. This effect will be most pronounced under conditions of Se deficiency, when the percent of Cys insertion into SelP is higher. It appears that, depending on the abundance of the non-Sec fraction, the levels of Se provided by SelP may be significantly overestimated in nutrition research. This raises issues with regard to the analysis and use of SelP as a marker Se status in human populations. Quantitative assessment of the degree of Sec replacement with Cys in human subjects differing in genotype, diet, disease status, sex and age would be informative for further application of this major Se biomarker.

## Supporting Information

S1 Fig Isolation of SelP from human plasma. A plasma pool of 5 human donors was purified by affinity column isolation. SelP was eluted with citric acid (50 mM, pH 2.0). (A) Elution profile indicating protein-containing fractions. Eluate fractions E2-E6 were pooled. (B) Western Blot analysis of the eluate-pool yields SelP bands in the expected size range.
(DOCX)
