# Supplementary material for: Regulation of Selenocysteine Content of Human Selenoprotein P by Dietary Selenium and Insertion of Cysteine in Place of Selenocysteine
Source: PLoS One. 2015 Oct 9;10(10):e0140353. doi: 10.1371/journal.pone.0140353 (PMC4599804; doi:10.1371/journal.pone.0140353)
Supplement: S1 Fig — A plasma pool of 5 human donors was purified by affinity column isolation. SelP was eluted with citric acid (50 mM, pH 2.0). (A) Elution profile indicating protein-containing fractions. Eluate fractions E2-E6 were pooled. (B) Western Blot analysis of the eluate-pool yields SelP bands in the expected size range. (DOCX) [file pone.0140353.s001.docx]

**Supplementary Information**


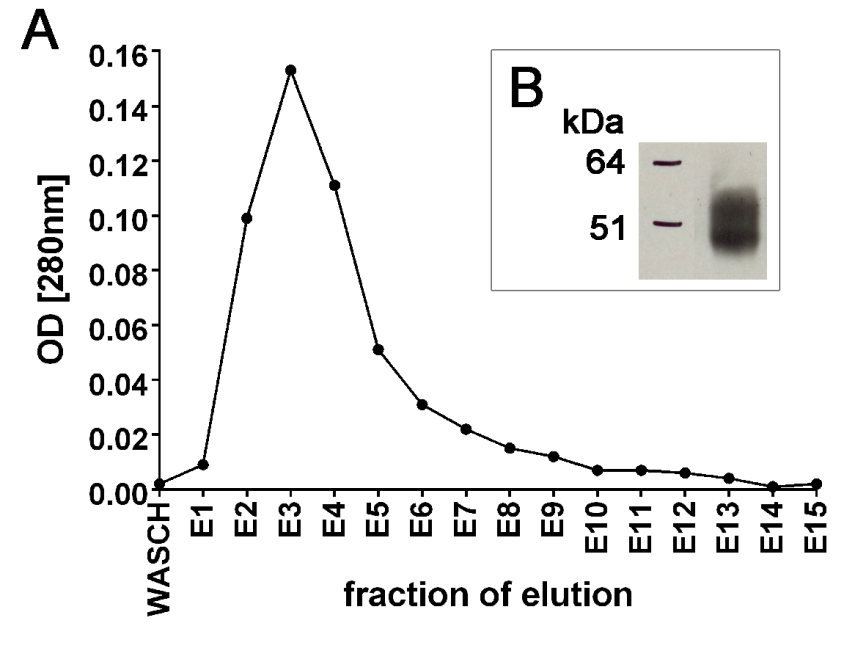


**S1 Fig. Isolation of SelP from human plasma.** A plasma pool of 5 human donors was purified by affinity column isolation. SelP was eluted with citric acid (50 mM, pH 2.0). **A.** Elution profile indicating protein-containing fractions. Eluate fractions E2-E6 were pooled. **B.** Western Blot analysis of the eluate-pool yields SelP bands in the expected size range.
